# In vivo anti-ulcer, anti-stress, anti-allergic, and functional properties of Gymnemic Acid Isolated from *Gymnema sylvestre* R Br

**DOI:** 10.1186/1472-6882-14-70

**Published:** 2014-02-22

**Authors:** Lilly Baptista Arun, Aarrthy M Arunachalam, Kantha Deivi Arunachalam, Sathesh Kumar Annamalai, Kalaivani Amit Kumar

**Affiliations:** 1 Center for Environmental Nuclear Research, Directorate of Research, SRM University, Kattankulathur, Chennai, Tamil Nadu 603203, India; 2 Kaplan University (Medical), Washington DC 20036, USA; 3 SRM Medical College Hospitals and Research Centre, SRM University, Kattankulathur, Chennai, Tamil Nadu 603203, India

**Keywords:** *Gymnema sylvestre*, Gymnemic acid, Anti- stress, Anti-ulcer, Anti- allergic, Antioxidant activities, Leaf extract

## Abstract

**Background:**

*Gymnema sylvestre* is a highly valued ethno pharmacologically important medicinal plant used currently in many poly-herbal formulations due to its potential antidiabetic activity and other health benefits. The present study was carried out to analyze the anti-stress, anti-allergic, and antiulcer activity of the bioactive compounds present in *Gymnema sylvestre* leaves.

**Methods:**

The preliminary phytochemical screening for bioactive compounds from aqueous extracts revealed the presence of alkaloids, triterpenes, flavonoids, steroids, and saponins. The antioxidant activities were investigated using DPPH radical scavenging method. The characterization of the extract was carried out using standard compound by High Performance Thin Layer Chromatography (HPTLC) and phytochemical analysis in terms of total phenol, total flavonoids, reducing power and antioxidant potentials, etc. The i*n vivo* studies on albino mice proved the purified fraction has anti-stress/anti-allergic activity against milk induced leucocytosis/eosinophilia and able to inhibit the aspirin induced gastric ulcers.

**Results:**

The quantitative estimation for aqueous extract exhibited total antioxidant (9.13 ± 0.04 μg/g), flavonoids (125.62 ± 26.84 μg/g), tannin (111.53 ± 15.13 μg/g), total phenol content (285.23 ± 1.11 μg/g) and free radical scavenging (52.14 ± 0.32%). Further the aqueous extract was consecutively purified by TLC and silica column chromatography. The purified fractions were characterized by HPTLC and GC-MS and the component was identified as gymnemic acid. The potency of the antimicrobial activity of the extract was studied with bacteria. Pharmacological experiments clearly demonstrated that the extracts of all plants given orally showed significant gastric protection against the asprin-induced gastric ulcer model in mice. Furthermore, healing effects were also confirmed through histopathological examination.

**Conclusions:**

The aqueous extracts of the leaves of *Gymnema sylvestre* possess anti ulcerogenic, Anti allergic, Anti stress, properties that may be due to cytoprotective mechanism. These results support the ethno medical uses of the plant in the treatment of gastric ulcer.

## Background

Plants have always been a prototypical source of drugs and many of the formerly available drugs have been derived directly or indirectly from them. A wide array of plant derived active principles representing numerous chemical compounds has demonstrated activity consistent with their possible use in the treatment of several diseases [1]. In Recent years, the use of ethno botanical information in medicinal plant research has gained considerable attention in segments of the scientific community [2]. In one of the Ethno botanical survey of medicinal plants commonly used by Kanitribals in Tirunelveli hills of Western Ghats in Tamil Nadu, India, has revealed that *Gymneme sylvestre* as the most important species according to their use [2].

The use of plant parts and isolated phytochemicals for the prevention and treatment of various health ailments has been in practice from time immemorial [3]. One of such plant is *Gymnema sylvestre* R. Br., commonly known as 'Meshasringi' which is distributed over most of India and it has a reputation in traditional medicine as a stomachic, a diuretic and as a remedy to control diabetes mellitus. *Gymnema sylvestre* R. Br [4] is a woody, climbing plant that grows in the tropical forests of central and southern India and in parts of Asia [5]. It is a pubescent shrub with young stems and branches, and has a distichous phyllotactic opposite arrangement pattern, which are 2.5-6 cm long and are usually ovate or elliptical, the flowers are small, yellow, in umbellate cymes and follicles are terete, lanceolate, up to 3 inches in length [6].

*Gymnema sylvestre* has been used in the treatment of diabetes since ages in folk, ayurvedic and homeopathic systems of medicine [1,4,7]. It is also used in the treatment of asthma, eye complaints, family planning, snakebite, urinary complaints, stomach ailments, piles, chronic cough, breathing troubles, colic pain, cardiopathy, constipation, dyspepsia and hemorrhoids, hepatosplenomegally [8]. In addition, it also possesses antimicrobial [9], antitumor [5], obesity [10], anti-Inflammatory [11], and Anti-hyperglycemic Activity [12].

Since there is an increase in the use of *G. sylvestre* as an alternative medicine, it has become even more important to analyze these parameters to give a contribution to the valorization of this natural source [13]. *G. Sylvestre* is used in several commercial formulations viz. Madhu Rakshak, Nature care Gymnema (Dabur India Ltd., New Delhi, India), Dolabi (Hamdard Laboratories, New Delhi, India), Blood sugar (Nutrasanus), Glucose Support (Vitabse, Monroe), Nutrilite (Amway Pvt. Ltd.) Dibecon, Ayurslim, Meshashringi (Himalaya Drug Co., Bangalore, India) and many others [13,14].

Even though many commercial drugs are available till date no reports are available on the anti-stress, anti-allergic, and antiulcer activity of the plants extracts so the present study was performed using various *in vivo* systems to explore the underlying mechanisms and mode of action. Since some researchers reported the seasonal variation of bioactive ‘gymnemagenin’ in *Gymnema sylvestre*[13,15]*,* so it is indispensable to characterize the phytochemical constituents of the collected plant for our study. So initially we have characterized the phytochemical constituents of the leaf extract and characterized for the pharmacological properties.

## Methods

Fresh leaves of *G. sylvestre* from plants of same age group from a single population were collected from experimental Herbal Garden, Tamil University, Thanjavur, Tamil Nadu, India in July, 2010. The herbarium was prepared for authentication (Ref. No: SRM\CENR\PTC\2010\03), and taxonomic identification was done by Dr. Jayaraman, Professor, Department of Botany, Madras Christian College, Tambaram, Chennai, Tamil Nadu and maintained in research laboratory for further reference.

### Preparation of aqueous extract

The leaves of *G. sylvestre* were first washed with distilled water to remove the dirt and further washed with mild soap solution and rinsed thrice with distilled water. The leaves were blotted with tissue paper and shade dried at room temperature for at least 2 weeks. After complete drying, the leaves were cut into small pieces and powdered in a mixer and sieved using a 20 μ mesh sieve to get a uniform size range for use in further studies. The 20.0 g of the sieved leaf powder was added to 100 mL of sterile distilled water in a 500 mL Erlenmeyer flask and boiled for 5 minutes. The flasks were kept under continuous dark conditions at 30°C. The extract was filtered and stored in an airtight container and protected from sunlight for further use [16,17].

### Preliminary phytochemical activity

The qualitative phytochemical analysis of *G. sylvestre* extracts were performed following the methods of Parekh and Chanda [18] to determine the presence of alkaloids (Mayer’s, Wagner, Dragendorff’s test), flavonoids (alkaline reagent, Shinoda), phenolics (lead acetate, alkaline reagent test), triterpenes (liberman-burchard test), saponins (foam test), tannins (gelatine). The results were qualitatively expressed as positive (+) or negative (−) [19].The chemicals used for the study were purchased from Sigma-Aldrich (Chennai, India).

### Quantitative phytochemical analysis

#### Estimation of total phenolic content

The total phenolic content of each extract was measured using an adapted Folin Ciocalteu colorimetric method. About 200 μl of 10% (v/v) Folin Ciocalteu reagent was mixed with 100 μl of aqueous extract in phosphate buffer (75 mM, pH 7.0). Gallic acid as a positive control and phosphate buffer as a negative control and the absorbance was measured at 765 nm after 30 minutes of incubation using UV-Vis 3000+ double beam spectrophotometer (Lab India, Maharashtra, India). A standard curve was calculated using gallic acid concentrations ranging from 0.05 to 0.5 mM and the results were expressed as mg/g gallic acid equivalents (GAE) of dried weight [20,21]. All the experiments were carried out in triplicate and the results were averaged to express as mean ± SD.

#### Estimation of total flavonoids

The aluminum chloride colorimetric method was used for flavonoid determination. 250 μl of each sample was mixed with 1.25 ml of deionized water and 0.075 ml of 5% sodium nitrite. After 6 min, 0.15 ml of 10% aluminum chloride was added and after another 6 min the product was mixed with 0.5 ml of 1 M sodium hydroxide and 2.5 ml of deionized water. Total flavonoids were measured at 510 nm using UV-Vis 3000+ double beam spectrophotometer (Lab India, Maharashtra, India). The results were given in mg CE/ g plant extract of cathequin equivalent [22,23].

#### Total antioxidant capacity

For total antioxidant capacity assay, 0.3 ml of the gymnema extract (10 mg/ml) dissolved in water was mixed with 3 ml of reagent solution (0.6 M sulfuric acid, 28 mM sodium phosphate and 4 mM ammonium molybdate). The reaction mixture was incubated and absorbance was measured at 695 nm against reagent blank. Gallic acid was used as the standard and the total antioxidant capacity was expressed as equivalents of ascorbic acid [23,24].

#### DPPH radical scavenging assay

The method of Blios was used for the determination of scavenging activity of the DPPH (2, 2-Diphenyl-1-Picrylhydrazyl) free radical. The reaction mixture (DPPH and extract) was vortexed, incubated and its absorbance was measured at 517 nm. The scavenging ability of the plant extract was calculated using the following equation:

$$\begin{array}{l} \text{DPPH}\;\text{Scavenging}\;\text{activity}\left(\%\right)\\ \;=\left[\left(\text{Abs}\;\text{control}–\text{Abs}\;\text{sample}\right)\right]\times 100\left(\text{Abs}\;\text{control}\right)] \end{array}$$

Where, *Abs control* is the absorbance of DPPH without sample; *Abs sample* is the absorbance of DPPH with sample [25].

### Purification and characterization of *G. sylvestre* extract

#### HPTLC screening

High Performance Thin Layer Chromatography is a planar chromatography where the separation of the sample components is achieved on high performance layers with detection and acquisition using an advanced workstation. Camag HPTLC System, equipped with a Linomat V with Camag 100 μl syringe sample applicator, a twin chamber tank, a model CAMAG Twin through glass chamber (20 × 10) Thin Layer Chromatography (TLC) scanner and Camag TLC scanner III software Win Cats 4.03 version were used in the study. TLC Aluminum sheets (20 cm × 10 cm) of silica gel GF 254 were used. A 20 μl reference standard gymnemic acid (1 μg/μl stock solution) of 92% purity prepared in methanol were applied to the TLC plate. 20 μl extract of each sample was applied to TLC plate. Three identical plates were prepared for concurrent results. The plates were developed up to 80 nm under chamber saturation conditions. After air drying the solvent, the plates were scanned using scanner III at 290 nm wavelength in absorbance mode [26,27].

#### Gas chromatography analysis

Analysis was carried out in a Agilent gas chromatograph fitted with a 6890 N Model fused silica column HP-5MS silica column (Agilent J&W Gc column) 30 Length (m), 0.250 Diam. (mm), 0.25 Film (μm), interfaced with an mass selective detector 5973B inert XL MSD operated by K. Hari Chandra Prasad using Chemstation software. Analytical conditions were injector and transfer line temperatures 250°C and 280°C, respectively, oven temperature was programmed at 60°C (isothermal for 5 min), with an increase of 4°C/min to 130°C (isothermal for 10 min), then 4°C/min to 240°C; the carrier gas used was helium at 1 ml/min; injection of 2 μl (10% hexane solution); split ratio 1:50 whereas split flow was 50 ml/min; standard electronic impact (EI) MS source temperature was 230°C; MS quadruple temperature was 150°C; mass scan range was 30-550 amu at eV; scan velocity was 1.22 scans s^−1^; and the resulting EM voltage was 2000 V [3,4,28].

#### Antimicrobial activity

As reported by our earlier works [16,19]*,* antimicrobial activity tests were carried out by disc-diffusion method using 100 μl of suspension containing 10^8^ CFU/ml of bacteria on Muller Hington Agar (MHA). The 6 mm in diameter discs were impregnated with 10 μl of the extracts (300 μg/disc) at the concentration of 30 mg/ml and placed on the clinical pathogens inoculated agar plates. The negative controls were prepared using the same solvents employed to dissolve the plant extracts. The standard drugs ofloxacin (10 μg/disc), Ciprofloxacin (10 μg/disc), netilmicin (30 μg/disc) were used as positive reference standards to determine the sensitivity of one strain/isolate in each microbial species tested. The inoculated plates were incubated at 37°C for 24 h for clinical bacterial strains growth. Antimicrobial activity was evaluated by measuring the zone of inhibition against the test organisms in duplicate. The antimicrobial activity of *Gymneme sylvestre* extract was evaluated against the growth of *Staphylococcus aureus, Bacillus cereus, Pseudomonas aeruginosa, Escherichia coli, and Strepococcus pyogenes*[29,30]*.*

### Pharmacological properties

#### *In vivo* studies

Swiss albino male mice weighing 25 g – 30 g obtained from Animal House, SRM Medical College Hospital & Research Centre, SRM University were used in this study. The animals were housed in polypropylene cages (47 cm × 34 cm × 18 cm), lined with husk which were renewed every 24 hrs. The animals were fed on a standard pellet diet and water *ad libitum* throughout the experiment. The experimental animals were maintained in a controlled environment (12:12 hr light and dark cycle) and temperature (24°C ±2°C). The experiments were carried out in accordance with the guidelines of the Committee for Control and Supervision of Experiments on Animals (CPCSEA), New Delhi, India and the experimental protocol was approved by the Ethical Committee for Animal Experimentation (Ethical clearance number: 14/IAEC/10) of SRM University. The animals were acclimatized for one week before starting the experiments.

#### In vivo assessment of anti-stress (adaptogenic activity)

Mice were divided into six groups (n = 6). Group I received vehicle, 5% polyethylene glycol (5 ml/kg, i.p.); group II was treated with milk (4 ml/kg, i.p.); test groups III– VI were treated with different concentration of *G. sylvestre* extract (50-200 mg/kg, i.p.) respectively and after 1 hr of drug treatment each animal was injected with pasteurized milk (4 ml/kg, i.p.). Total leukocyte count was checked for each group before treatment and also 24 hrs after milk injection. Blood samples were collected from retro-orbital plexus. Total leukocyte count was also checked in each group before drug administration and 24 hrs after milk injection. Blood was sucked in WBC pipette up to mark and kept aside for 5 min. Total leukocyte and eosinophil counts were taken in each group before drug administration and 24 h after milk injection. Difference in total leukocyte and eosinophil count before and 24 h after drug administration was calculated [31].

### Anti-allergic activity –Milk induced eosinophilia in mice

Eosinophilia was induced in mice using milk as per the protocol described below. Mice were divided into six groups of six animals in each group. Blood was collected from retro-orbital plexus. Group I received vehicle, 5% polyethylene glycol (5 ml/kg, i.p.); group II served as negative control and treated with milk (4 ml/kg,i.p.); test groups III– VI were treated with different concentration of *G. sylvestre* extract (50-200 mg/kg, i.p.) respectively and after 1 h of drug treatment each animal was injected with boiled and cooled milk (4 ml/kg, i.p.). The eosinophil count was done in each group before treatment and 24 h after milk injection and the blood samples were collected from retro-orbital plexus. The eosinophil count was done in each group before drug administration and 24 h after milk injection. The blood was sucked in WBC pipette and diluted with eosin stain. The eosin solution facilitates destruction of all corpuscles except eosinophil. Neubaur’s chamber was charged with above fluid and eosinophilis were counted. The difference in eosinophil count before and after 24 h of the treatment was calculated [32,33].

### Antiulcer activity - Aspirin-induced ulcer assay

Thirty fasted mice were also used this model as five groups of six mice each. Groups I and II of this model received distilled water (2 ml/kg) and omeprazole 20 mg/kg p.o respectively, while groups III, IV, V and VI received 50, 100,150 and 200 mg/kg p.o of the extract. Animals in all the groups were fasted for 18 h after the respective assigned treatment and were anaesthetized with anesthetic ether. The abdomen was opened by a small midline incision below the xiphoid process and pylorus portion of stomach was lifted out and ligated [34]. Precautions were taken to avoid traction to the blood supply. The stomach was sutured with interrupted sutures. Four hours after pylorus ligation the rats were sacrificed and the stomach was removed [35].

The stomach was then incised along the greater curvature and observed for ulcers. The numbers of ulcers were counted using a magnifying glass and the diameter of the ulcers were measured using vernier calipers. As reported by Dharmani et al, (2005) [36] arbitrary scoring system was used to grade the incidence and severity of lesions: (i) score 10 = denuded epithelium; (ii) score 20 = petechial and flank haemorrhages; (iii) score 30 = one or two ulcers; (iv) score 40 = multiple ulcers; (v) score 50 = perforated ulcer.

Ulcer index (UI) was then calculated from the above scorings as reported by Umamaheswari et al, (2007) [37].

$$\mathrm{UI}=U_{N}+U_{s}+U_{p}\times 10^{-1}$$

Where,

Where, U_N_ is the average of number of ulcers per animal, U_S_ is the mean severity of ulcer score and U_p_ is the percentage of animals with ulcer incidence [37].

### Histopathological evaluation

Stomachs were immersed in a 10% formalin solution for histopathological examination following the assessment of ulcer. The gastric tissue samples were fixed in neutral buffered formalin for 24 h. Sections of tissue from stomachs were examined histologically to study the ulcerogenic and or anti-ulcerogenic activity of *G. sylvestre*. The tissues were fixed in 10% buffered formalin and were processed using a tissue processor. The processed tissues were embedded in paraffin blocks and about 5-μm thick sections were cut using a rotary microtome. These sections were stained with hematoxylin and eosin using routine procedures. The slides were examined microscopically for morphological changes such as congestion, hemorrhage, edema, and erosions using an arbitrary scale for the assessment of severity of these changes [19,35,37].

### Statistical analysis

All the assays were conducted in replicates and data were expressed as mean ± SD. The statistical significance was calculated using one-way ANOVA followed by Dunnet comparison test. P values <0.05 were considered significant.

## Results and discussion

### Phytochemical screening

The results of the preliminary phytochemical screening of Aqueous extracts of *G. sylvestre* revealed the presence of alkaloids, phenols, flavonoids, sterols, tannins and triterpenes (Table 1). As tabulated in the Table 2 the total flavonoids were 125.62 ± 26.84 μg/g, total phenol content was 285.23 ± 1.11 μg/g and tannin 111.53 ± 15.13 μg/g were present in the water extract of *G. sylvestre.* The flavonoids and phenolic compounds exhibited a wide range of biological activities like antioxidant and lipid peroxidation inhibition properties.

**Table 1:** **Phytochemical screening of leaf extract of *G. sylvestre***

**Compound**	**Aqueous extract**
Alkaloids	+
Triterpenoids	+
Glycosides	-
Saponins	+
Tannins phenols	+
Flavonoids	+
Steroids	+

+Present. -Absent.

**Table 2:** **Estimation of phytochemical compounds of leaf extract of *G. sylvestre***

**Bioactive compounds**	**(μg/g)**
Total antioxidant^a^	9.13 ± 0.04
Flavonoids^b^	125.62 ± 26.84
Tannin^c^	111.53 ± 15.13
Total phenol content^d^	285.23 ± 1.11
Free radical scavenging^d^	52.14 ± 0.32

All the values given in the table are means of triplicates determinations. Data presented as the mean ± standard deviation; ^a^Gallic acid equivalent; ^b^Tannic acid equivalent, ^c^Quercetin equivalent, ^d^Catechin equivalent.

This was proved by testing the total antioxidant 9.13 ± 0.04 μg/g and DPPH radical scavenging assay 52.14 ± 0.32% respectively (Table 2). All these results indicate flavonoids could be a significant source of antioxidant property but, the activity mostly depends upon their molecular structure and position of hydroxyl groups.

### Characterization of *G. sylvestre* aqueous extract

**Figure 1:**
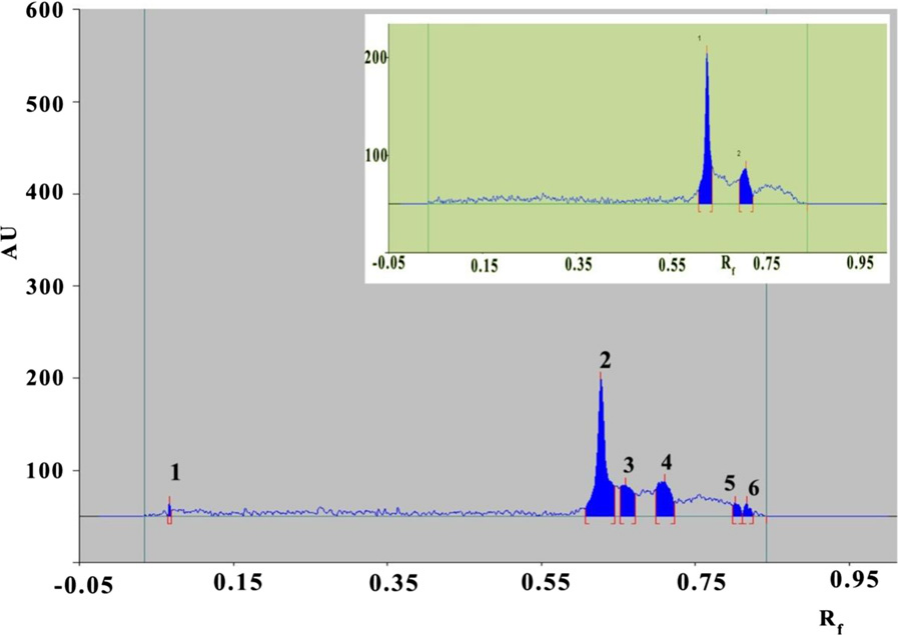
**HPTLC chromatograms of Aqueous extract of *G. sylvestre *** **insert within image reference standard gymnemagenin.**

By testing different mobile phases for the separation of extracts of leaves of *G. sylvestre* by HPTLC, the desired resolution of gymnemagenin with symmetrical and reproducible peaks was achieved using n-butanol: acetic acid: water (6:3:1) as the mobile phase. The chromatograms of standard and of the leaf extract of *G. sylvestre* are shown in Figure 1 respectively.

From the Figure 1 it is observed that the calibration curve was linear; the correlation coefficient indicated good linearity between concentration and area. To ascertain peak purity of test sample, we compared in vivo and in vitro methanol extract reflectance spectra with standard, which provides test sample purity as shown in Figure 1. When applied to leaf extracts of *G. sylvestre*, the content of gymnemagenin was found to be 2.34% dry weight. Furthermore, a correlation coefficient value of 0.99 indicates good linearity between concentration and area. Using the proposed HPTLC method, the Rf of gymnemagenin was found to be 0.63. The chromatograms of the reference standard gymnemagenin are shown as an insert and that of the aqueous extract.

**Figure 2:**
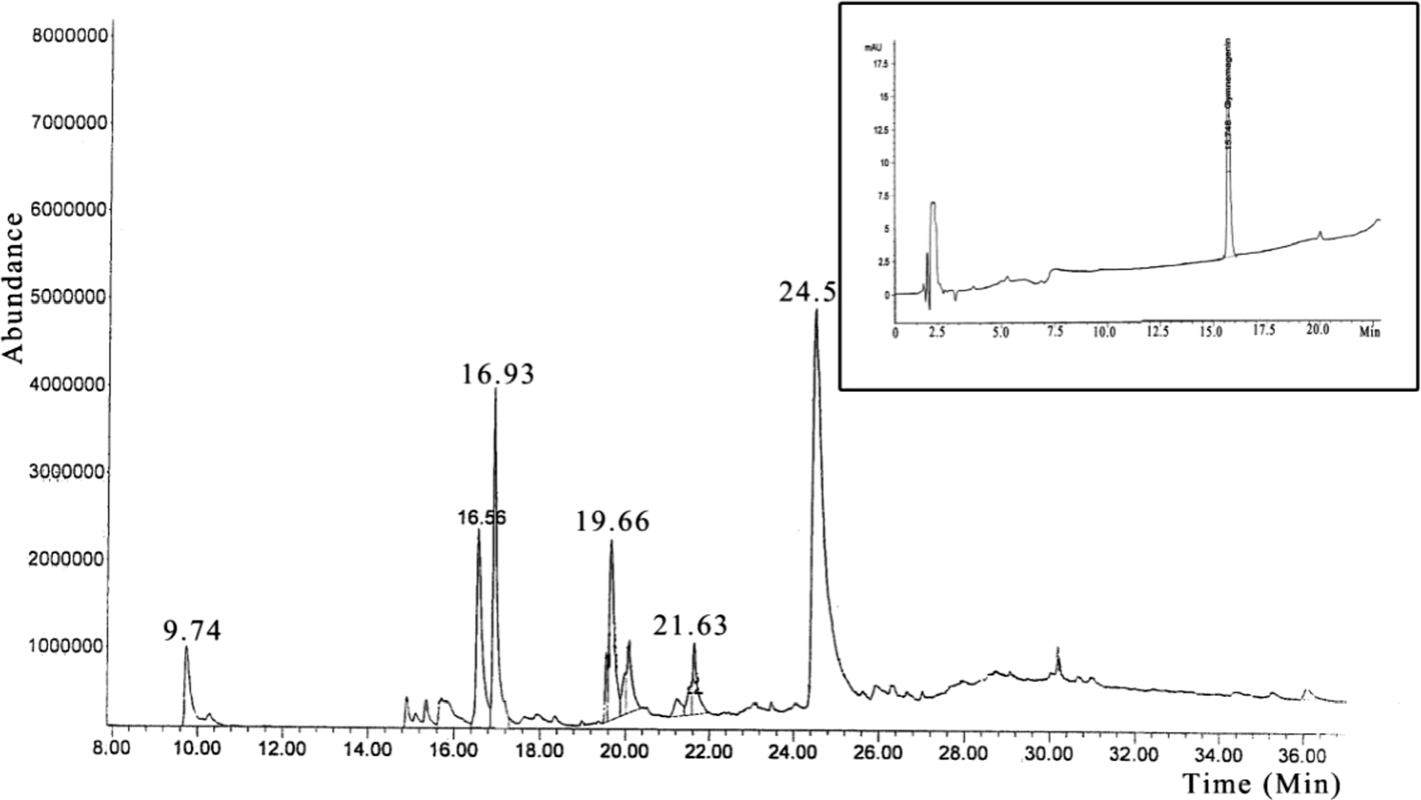
**Gas Chromatogram of *G. sylvestre *** **insert within image GC Chromatogram of the standard gymnemagenin.**

A representative chromatogram of the aqueous extract of *G. sylvestre* leaves is shown in Figure 2 and indicated the presence of 18 components. These assignments of gymnemagenin were supported by comparison of their linear retention indices calculated using standard with those reported in the literature [5,38,39]. The GC spectrum confirmed the extracted bioactive molecule as gymnemic acid and the inserts with in the image shows the standard gymnemagenin.

### Antimicrobial activity

**Figure 3:**
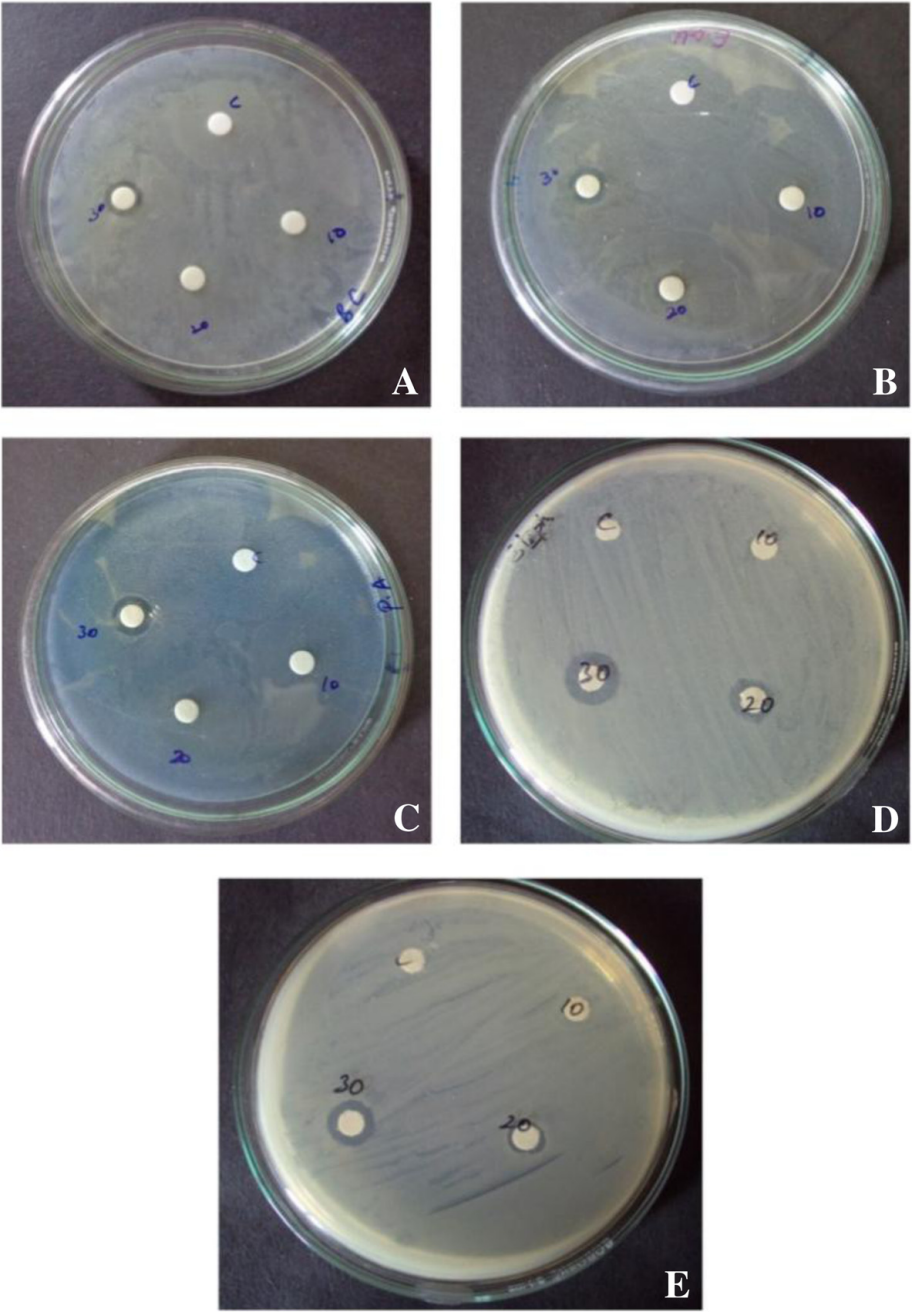
**Zone of inhibition of the aqueous extracts of *Gymnema sylvestre *** **against the pathogens A) *Bacillus cereus *** **B) *Escherichia coli *** **C) *Staphylococcus aureus *** **D) *Pseudomonas aerogenosa *** **E) *Streptococcus pyogenes *** **by disc diffusion method.**

The antimicrobial activities of *G.sylvestre* extracts against clinical pathogens were quantitatively assessed by the zone of inhibition for the pathogens *Staphylococcus aureus, Bacillus cereus, Pseudomonas aeruginosa, Escherichia coli* and *Strepococcus pyogenes. T*he calculated diameters of the zones of inhibition are tabulated in Table 3 and the zones were clearly observed from the Figure 3.

**Table 3:** **In vitro antibacterial screening of aqueous extract of *Gymnema sylvestre***

**Selected microbial strains**	**Zone of inhibition (mm)**		
	**10 mg/ml**	**20 mg/ml**	**30 mg/ml**
*Bacillus cereus*	-	9 ± 0.22	11 ± 0.52
*Escherichia coli*	8 ± 0.3	9 ± 0.41	12 ± 0.21
*Staphylococcus aureus*	-	12 ± 0.28	15 ± 0.32
*Pseudomonas aerugenosa*	-	9 ± 0.34	13 ± 0.41
*Streptococcus pyogenes*	-	11 ± 0.41	13 ± 0.57

The values were means ± standard deviation of three replicate experiments. The strain used for the study was obtained from MTCC.

Our results showed the inhibition of growth for both Gram-positive and Gram-negative organisms by the plant extracts. As reported by Benz & Bauer (1988), [40] the gram-negative bacteria possess a hydrophilic outer membrane, due to the presence of lipopolysaccharide molecules; so the small hydrophilic solutes of the plant extracts can pass the outer membrane through abundant porin proteins which provide the hydrophilic trans-membrane channels. Whereas the outer membrane serves as a penetration barrier towards macromolecules and to hydrophobic compounds. So the bioactive compounds has crossed the membrane and inhibited the growth of the selected pathogens.

This antimicrobial activity is related to the different compounds present in the secondary metabolites such as alkaloids, phenols and terpenoids reported or identified in these plant species which needs further investigations. The antimicrobial activity of the tested plant extracts against the gram negative bacteria was either low or inactive. In general, Gram positive bacteria show higher resistance towards antimicrobial agents and this is evident from the susceptibility results of *Staphylococcus aureus.*

### Anti-stress activity of *G. sylvestre*

Stress basically is a reaction of mind and body against changes in the homeostasis [41]. It is a physiological condition caused due to trauma, polluted air exposure, radiation, reactive nitrogen and oxygen species, which lead to immunodeficiency and oxidative stress [42]. The increase in white blood cells (leukocytes) count is a most reliable biomarker in assessing the stress levels in the organisms. The parenteral administration of milk produced a marked increase in the leukocytes count and this stressful condition can be normalized by administration of an anti-stress or adaptogenic drug [43]. The mice pre-treated with various concentrations of *G. sylvestre* extract showed reduction in leukocyte count when induced by milk, whereas negative control failed to reduce leukocyte count significantly (Table 4).

**Table 4:** **Effect of leaf extract of *G. sylvestre *** **on milk-induced leukocytosis and eosinophil in mice**

**Group**	**Treatment**	**Total leucocytes count**	**Eosinophil count**
I	5%PEG	121.23 ± 13.15	53.0 ± 19.34
II	milk	3180.21 ± 136.0	84.1 ± 42.90
III	50 mg/kg	2025.03 ± 272.87	66.7 ± 16.67*
IV	100 mg/kg	2483.34 ± 148.14*	66.6 ± 10.54*
V	150 mg/kg	1766.67 ± 316.14*	50.0 ± 12.91*
VI	200 mg/kg	1545.89 ± 732.49*	37.8 ± 74.11

Data is presented as mean ± SEM; *< 0.05 compared with vehicle + milk group (group II; negative control).

The treated groups (IV-VI) showed significant reduction of leukocytes counts in linear fashion with increasing concentration (p < 0.05). Thus *G. sylvestre* extract showed protective effect against milk-induced leucocytosis in mice. These signs represent some of the core symptoms observed in depressed patients or in individuals under intense stress. Similar activity was reported from the extracts of *Abrus precatorius*[32], *Alchornea cordifolia*[44] and *Evolvulus alsinoides*[45].

### Anti-allergic activity of *G. sylvestre*

The eosinophils are reported to mediate inflammatory and cytotoxic events associated with allergic disorders, including bronchial asthma, rhinitis and urticaria [46]. As shown in table (4), the administration of boiled and cooled milk in group (II) showed an abnormal increase in eosinophil count compared to the group (I). Wechsler (2007) [47] eported that, increase in peripheral eosinophil to more than 4% of total leucocyte countr is directly associated with respiratory disorder and are often allergic in nature. The mice pretreated with various *G. sylvestre* extracts (group III-VI) showed significant (p < 0.05) reduction in eosinophil count induced by milk. So, as reported by Brahmans and Dardymov,1969 [48] the most important characteristic of an adaptogen, is its ability to increase resistance to adverse influences of a wide range of physical, chemical and biological factors; irrespective of the previous pathologic condition. In this circumstances *G. sylvestre* extracts significantly reduced eosinophils, which shows that these extracts are useful in allergic and asthmatic conditions.

Anti-allergic activity of *G. sylvestre* may be due to the presence of tannins (111.53 μg/g), total phenols (285.23 μg/g) and flavonoids(125.62 μg/g). In conclusion, aqueous extract of *G. sylvestre* has anti-allergic activity and potential to stabilize mast cells by antagonizing the milk-induced eosinophilia. As reported by Chen et al., 2013 [49], the phenolic compounds has the anti-allergic effects could be partly mediated through the reduction of Ca2+ influx and elevation of cAMP in the mast cells. Similarly the G.sylvestre extract has the higher phenolic content (285.23 μg/g) which could be the compound which induces the anti- allergic effects.

### Antiulcer activity of *G. sylvestre* extract

The antiulcer activity of *G. sylvestre* extract can be established in mice models in this study against aspirin induced ulcers. The negative control group showed a 9.7 ± 0.03 ulcer index, which happened due to the inhibition of prostaglandin synthesis, which is essential for mucosal integrity and regeneration, followed by reduction in mucosal blood flow and a consequent generation of ulcer [50]. The aqueous extract at all doses provided dose dependent protection and doses of 150 mg/kg and 200 mg/kg provided significant protection (89% and 100%, p < 0.05) when compared with the negative control (Table 5).

**Table 5:** **Effect of *G. sylvestre leaf *** **extracts on aspirin induced gastric ulcer in mice**

**Group**	**Treatment**	**Quantal ulcer incidence**	**Ulcer index**	% protection
I	Control	6/6	9.7 ± 0.03	--
II	Omeprazole- 20 mg/kg	6/6	3.5 ± 0.01	72%
III	50 mg/kg as extract	6/6	6.2 ± 0.05	44%
IV	100 mg/ kg as extract	6/6	5.9 ± 0.05	56%
V	150 mg/kg as extract	6/6	2.1 ± 0.02*	89%
VI	200 mg/kg as extract	6/6	0.5 ± 0.07*	100%

Data is presented as mean ± SEM; *< 0.05 compared with negative control.

**Figure 4:**
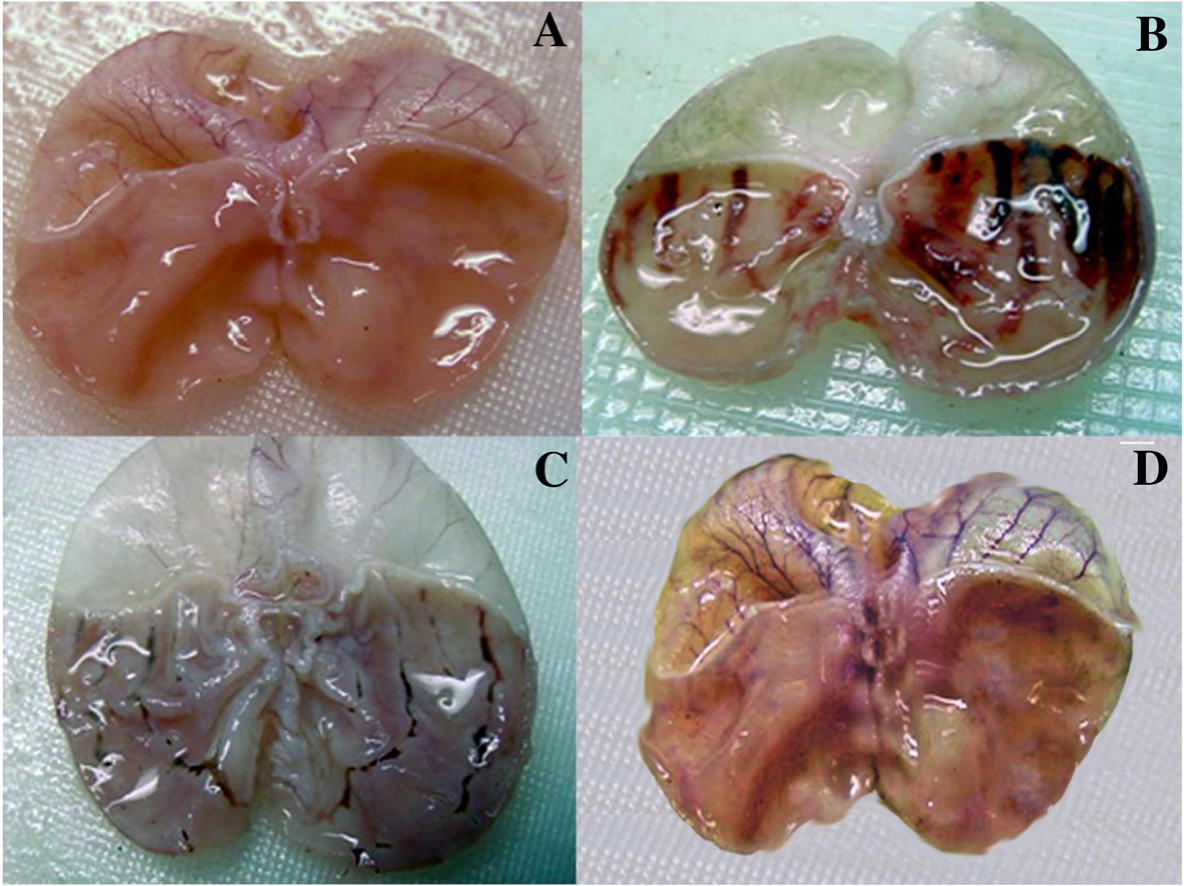
**Gross appearances of stomach (A) Control (B) Normal Group Ulcer Induced by Asprin (C) Omeprazol treated section (D) High concentration of *G. sylvestre *** **treated section.**

From the Figure 4 it is observed that the production of inflammatory mediators is also an important factor for the mechanisms of lesions. In addition, gastric blood flow stasis and micro vascular disruption bring about haemorrhage and necrotic tissue injury.

**Figure 5:**
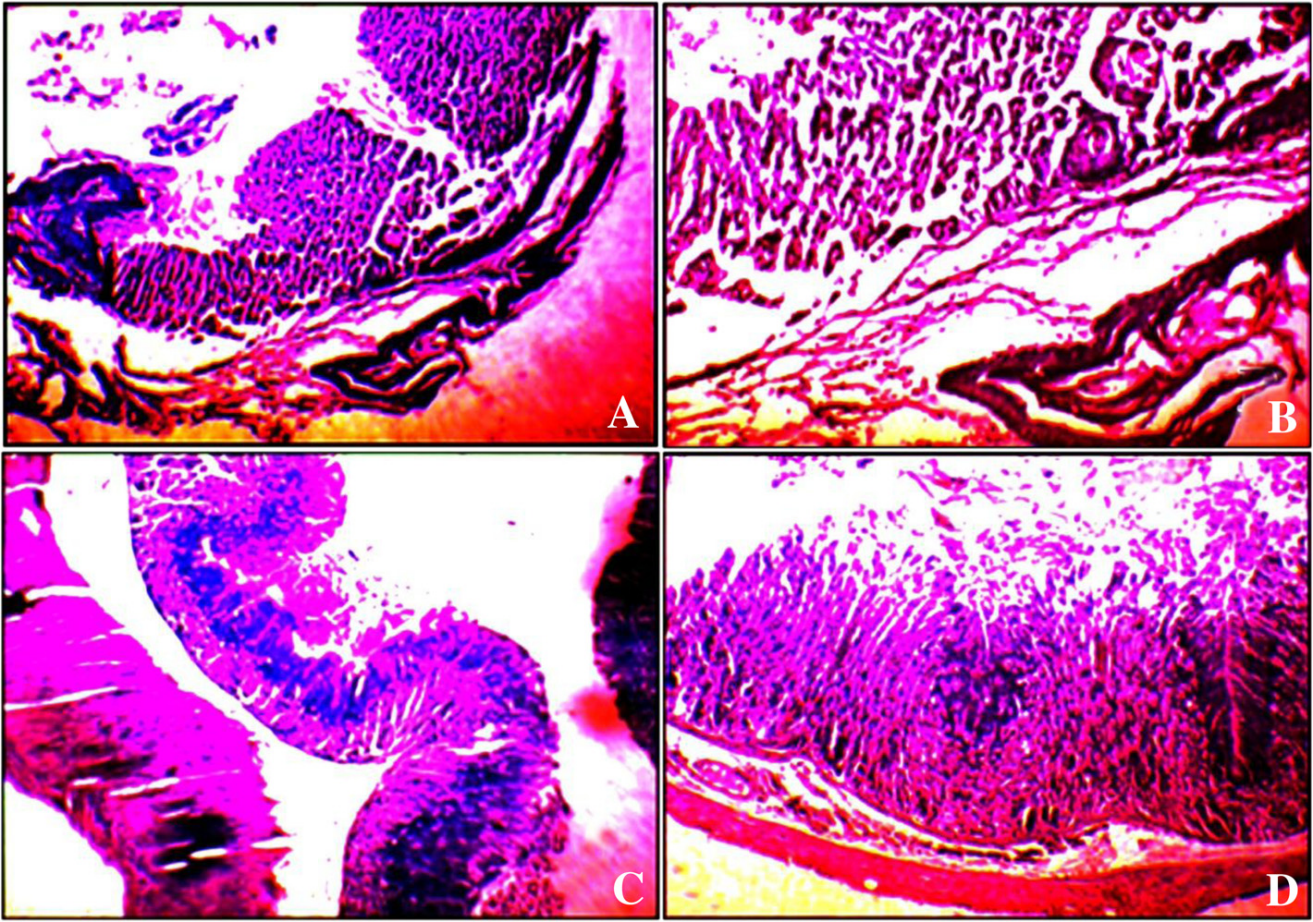
**Photomicrographs of HE staining of gastric mucosa (magnification × 100).** (Hematoxin&Eosin) **(A)** Control Group **(B)** Normal Group Ulcer Induced by Asprin **(C)** Omeprazol treated section **(D)** High concentration of *G. sylvestre* treated.

The histological studies of gastric mucosa of mice revealed a significant reduction in gastric erosion and lesions in *G. sylvestre* treated group, which is similar to that in the Omeprazol treated group, as compared to the control group (Figure 5).

As observed from the Figure 5 the intestinal metaplasia cannot always be objectively classified by histological methods, because it is sometimes difficult to recognize the brush border of absorptive cells using conventional haematoxylin–eosin (HE) staining. In the control group (Figure 5A), there is no macroscopic or microscopic lesions were observed. Compared to control group, intra gastric administration of aspirin induced macroscopic morphological changes, such as linear haemorrhages, and mucosal erythema in the mucosal layer were observed in the ulcer induced group (Figure 5B). Similar results were reported by Choi et al, 2010 [51] that the aspirin-induced rats showed predominant mucosal hyperemia and haemorrhagic lesions with edema covering the total glandular area of the stomach, and it was evident in indicating acute ulceration in the non-pretreated ulcerated mice. The mice treated with omiprazole (20 mg/kg) (Figures 5C), and *G. sylvestre* treated (Figure 5D) had considerably reduced areas of gastric damage formation compared with ulcer group. From our results that lesions on the gastric mucosa were significantly reduced in the animals pre treated with *G. sylvestre* at the doses of 100 mg/kg.

In control section (Figure 5A) the gastric mucosal layer sub mucosa shows inflammation mucosa is intact with sub mucosal chronic inflammatory infiltrate. In the aspirin treated group the (Figure 5B) section showed submucosal congestion edema, and high inflammation sub mucosal chronic inflammatory in filtrate. In the Omeprazole treated group (20 mg/kg) (Figure 5C) shows no significance change in histopathology which is almost appear normal.

Therefore, overall histological scores showed that pre-treatment with *G. sylvestre* against asprin induced ulcer revealed significant ulcer protection by efficient epithelialization, glandular organization, tendency of regeneration of mucosa and reduced size of ulcer crater. Our aqueous extract of *G. sylvestre* showed more protective effective as compared with the anti-ulcer properties of other medicinal plants such as Cassia sieberiana [52], *Simaba ferruginea*[53], *Voacanga africana*[54], *Cayratia trifolia*[55]*, Calotropis procera*[56]*, Cola cordifolia* bark and leaves [57], leaves of *Solanum torvum*[58]*and* red algae *Gracilaria changii*[59]*. G. sylvestre* is found to protect the aspirin induce ulcer at 100 mg/kg.

Al-Rejaie et al., 2012 [60] has reported that pre-treatment with G. sylvestre (100, 200, and 400 mg/kg) showed protection to the damaging action of ethanol induced gastric mucosal injury in rats. Also he has reported that even at 400 mg/kg only 63% protection was achieved against the ulcerogenic effect of ethanol. Since ethanol treatment itself significantly reduced the stomach proteins and nucleic acids contents of the animals by accumulating the toxic free radicals in the mucosal cells. But our results are promising that the aspirin induced ulcer is completely protected by 200 mg/kg of extract.

The phytochemical screening of the *G. sylvestre* extract showed the presence of flavonoids (Tables 1 and 2) which might be the reason for the observed antiulcer activity. Because flavonoids possess the ability to protect the gastric mucosa against variety of ulcerogenic agents due to their anti-inflammatory activity [61]. Moreover free radical-mediated stress has been one of the concerns in the pathogenesis of gastrointestinal disorders [62]. So the antioxidant activity of *G. sylvestre* extract (Table 2) has also been effective in preventing this kind of lesion because of their free radical scavenging properties.

## Conclusion

*Gymnema sylvestre* is a well-studied medicinal plant used since time immemorial. The wide varieties of compounds isolated from this plant were characterized for their structural, functional, and pharmaceutical properties using various *in vitro* and *in vivo* studies. This paper aims to detail some standard procedures to provide better scope for performing the anti-stress, anti-allergic and antiulcer properties of *G. sylvestre*. The plant is thus a promising source of anti ulcerogenic drug besides indication that gymnemagenin is one of the compounds responsible for these effects. Such findings are of extreme importance in the strive for future development of potent, safer and effective antiulcer agent. The different *in vivo* assays have once again proved this plant to be a potent source for treating several diseases.

## Competing interests

The authors declare that they have no conflict of interest.

## Authors’ contributions

LBA has performed the study; AMA has participated in the design of the study. KDA and SKA worked on the methods, data analysis, wrote the paper and revised it critically. KAK has performed the Histopathological evaluation. All authors read and approved the final manuscript.

## Pre-publication history

The pre-publication history for this paper can be accessed here:

http://www.biomedcentral.com/1472-6882/14/70/prepub
